# Ribosomal RNA regulates chromosome clustering during mitosis

**DOI:** 10.1038/s41421-022-00400-7

**Published:** 2022-05-31

**Authors:** Kai Ma, Man Luo, Guanglei Xie, Xi Wang, Qilin Li, Lei Gao, Hongtao Yu, Xiaochun Yu

**Affiliations:** 1 Westlake Laboratory of Life Sciences and Biomedicine, Hangzhou, Zhejiang China; 2 School of Life Sciences, Westlake University, Hangzhou, Zhejiang China; 3 Institute of Basic Medical Sciences, Westlake Institute for Advanced Study, Hangzhou, Zhejiang China; 4 Microscopy Core Facility, Biomedical Research Core Facilities, Westlake University, Hangzhou, Zhejiang China; 5 Institute of Biology, Westlake Institute for Advanced Study, Hangzhou, Zhejiang China

**Keywords:** Chromosome condensation, Chromosomes, Mitosis

## Abstract

Noncoding RNAs are known to associate with mitotic chromosomes, but the identities and functions of chromosome-associated RNAs in mitosis remain elusive. Here, we show that rRNA species associate with condensed chromosomes during mitosis. In particular, pre-rRNAs such as 45S, 32S, and 30S are highly enriched on mitotic chromosomes. Immediately following nucleolus disassembly in mitotic prophase, rRNAs are released and associate with and coat each condensed chromosome at prometaphase. Using unbiased mass spectrometry analysis, we further demonstrate that chromosome-bound rRNAs are associated with Ki-67. Moreover, the FHA domain and the repeat region of Ki-67 recognize and anchor rRNAs to chromosomes. Finally, suppression of chromosome-bound rRNAs by RNA polymerase I inhibition or by using rRNA-binding-deficient Ki-67 mutants impair mitotic chromosome dispersion during prometaphase. Our study thus reveals an important role of rRNAs in preventing chromosome clustering during mitosis.

## Introduction

Chromatin-associated RNAs play key roles in regulating numerous biological events, such as chromatin remodeling and transcription^1–4^. During mitotic prophase, chromatin undergoes condensation and forms highly compacted chromosomes. It is unclear whether the identities of chromatin-associated RNAs are altered during the chromatin condensation process.

Mitotic chromosomes are composed of several specific regions. The inner scaffold contains proteins, such as condensin, for maintaining the integrity of the condensed chromosomes^5,6^. The outer layer of condensed chromosomes, also known as the perichromosomal layer, includes a number of proteins and RNA species^7^. A recent analysis combining scanning electron microscopy with advanced proteomics indicates that the perichromosomal layer comprises 30%–47% of the entire chromosome volume^8^. Proteomics analysis shows that a number of proteins associate with the perichromosomal layer, and one prominent protein is Ki-67^9,10^.

Ki-67, a surrogate marker for cell proliferation, localizes in the nucleolus during interphase. Although Ki-67 may be involved in ribosomal biogenesis^11–13^, it is dispensable for the generation of mature rRNAs^14^. During mitotic prophase, the nucleolus is disassembled along with nuclear envelope breakdown^15^. Interestingly, Ki-67 is released before nucleolus disassembly and relocates to the perichromosomal layer of condensed chromosomes^13^. It has been shown that loss of Ki-67 depletes the mass of the perichromosomal layer and causes the coalescing of mitotic chromosomes^8,16^, suggesting that Ki-67 plays a key role to maintain the integrity of the perichromosomal layer. Ki-67 is a large nuclear polypeptide with several prominent domains. It has been shown that the C-terminal Leu-Arg rich (LR) domain recognizes chromosomal DNA and anchors Ki-67 on mitotic chromosomes, whereas the other regions of Ki-67 extend outwards to form the perichromosomal layer^16^.

In contrast to the protein components, mitotic chromosome-associated RNA species are much less studied. Since nucleolar proteins associate with small nucleolar RNA (snoRNA) to form RNPs, some snoRNA species, such as U3 snoRNA, are reported to be present at chromosome periphery^7,17,18^. In this study, we have carefully examined the chromosome-associated RNAs, and found that rRNAs are the major RNA species associating with condensed chromosomes. These rRNA species are evenly distributed in the perichromosomal layer of each chromosome via the interaction with Ki-67. These chromosome-associated rRNAs facilitate chromosome dispersion during prometaphase.

## Results

### rRNAs are associated with mitotic chromosomes

**Fig. 1:**
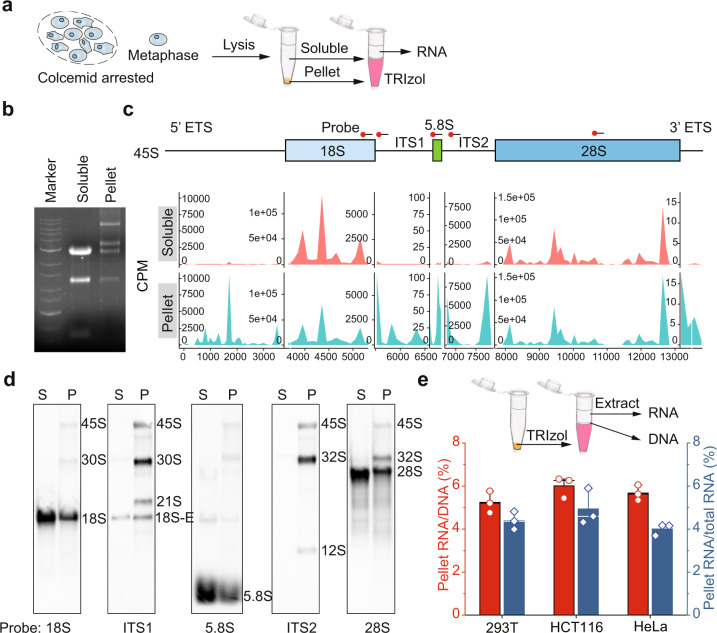
rRNA associates with chromosomes during mitosis. **a** A schematic diagram shows the procedure of RNA purification from soluble and pellet fractions of metaphase cells. **b** Purified RNA is analyzed by agarose gel. **c** Distribution of RNA-seq reads (CPM) across the full-length 45S pre-rRNA sequence in soluble and pellet total RNAs. **d** Northern blot analysis of the RNAs from the soluble (S) and pellet (P) fractions with probes targeting 18S, ITS1, 5.8S, ITS2, and 28S regions. The position of northern blot probes is shown in **c**. **e** The ratio of RNA in the pellets to total DNA mass (left *y* axis) and the ratio of RNA in the pellets to total RNA (right *y* axis).

To examine the chromosome-associated RNAs, we pretreated HCT116 cells with colcemid to arrest cells in prometaphase (Fig. 1a and Supplementary Fig. S1a). Mitotic cells were shaken off and lysed with NETN100 buffer (0.5% NP-40, 50 mM Tris-HCl, pH 8.0, 2 mM EDTA, and 100 mM NaCl), and both the NETN100 soluble fraction and the pellets were harvested (Fig. 1a). To validate the mitotic population, cells were examined by flow cytometry with Propidium Iodide and anti-histone H3pSer10 antibody. We found that all the cells were arrested at 4N stage, and majorities of cells were stained positively with anti-histone H3pSer10 antibody (Supplementary Fig. S1b). Moreover, we performed mitotic spreads and did not observe any interphase cells (Supplementary Fig. S1c). With western blotting, we detected cytoplasmic and nucleoplasmic proteins, including RPL3 and GADPH, in the NETN100 soluble fraction. In contrast, we did not find these representative proteins in the pellet fraction (Supplementary Fig. S1d), indicating that the NETN100 soluble fraction represents cytoplasmic/nucleoplasmic fraction of mitotic cells. In the pellets but not the NETN100 soluble fraction, we found nucleosomal histones, suggesting that components tightly associated with condensed chromosomes exist in the pellets, in agreement with earlier studies^19,20^. Next, we explored chromosome-associated RNAs in mitotic cells by isolating RNA from both the NETN100 soluble fraction and the pellets with TRIzol (Fig. 1a). In the cytoplasmic/nucleoplasmic fraction, two major bands were detected, correlating with the size of 28S and 18S rRNA. These results indicate the mature rRNA species in ribosomes existed in the cytoplasmic/nucleoplasmic fraction. Interestingly, in the chromosome fraction, we detected other RNA species with higher molecular weight in addition to 28S and 18S rRNA (Fig. 1b). To examine these RNA species, we performed RNA sequencing and found that these high-molecular-weight RNAs were pre-rRNAs, including 45S, 32S, and 30S pre-rRNA, because sequencing reads were highly enriched in the 5’ ETS and ITS1/2 regions of pre-rRNAs (Fig. 1c).

Transcription of pre-rRNAs is mediated by RNA polymerase I from rDNA loci during interphase. Immediately the following transcription, 47S pre-rRNA is digested into 45S pre-rRNA and further processed to produce mature rRNAs via complicated pathways^21^. To validate these chromosome-associated rRNAs, we designed probes covering every region of pre-rRNA for northern blotting (Supplementary Fig. S1e). We found various pre-rRNA species in northern blotting (Fig. 1d). In the pellet fraction, we also observed 18S, 5.8S, and 28S rRNAs that have the expected size of mature rRNAs. Since the final processing steps of these rRNA species are known to happen in the nucleolus^21^, it is possible that these chromosome-associated 18S, 5.8S, and 28S rRNAs are still part of the nucleolus. Next, using the same samples, we performed RT-qPCR with different sets of primers targeting rRNAs. Again, we found that pre-rRNAs, especially 45S, 30S, and 32S pre-rRNAs, were highly enriched in the chromosome fraction, mature 18S, 5.8S, and 28S rRNAs were present at relatively lower levels, compared to those in the soluble fraction (Supplementary Fig. S1e, f).

In addition, we examined the ratio between RNA and DNA on the mitotic chromosomes and found that chromosome-associated RNA was around 4%–5% of total RNA in different types of cells. Moreover, the chromosome-associated RNA was equivalent to 5%–6% of total genomic DNA mass in these cells (Fig. 1e). Thus, these results indicate that the majorities of pre-rRNA associate with condensed chromosomes during mitosis.

### rRNA localizes at the periphery of mitotic chromosomes

**Fig. 2:**
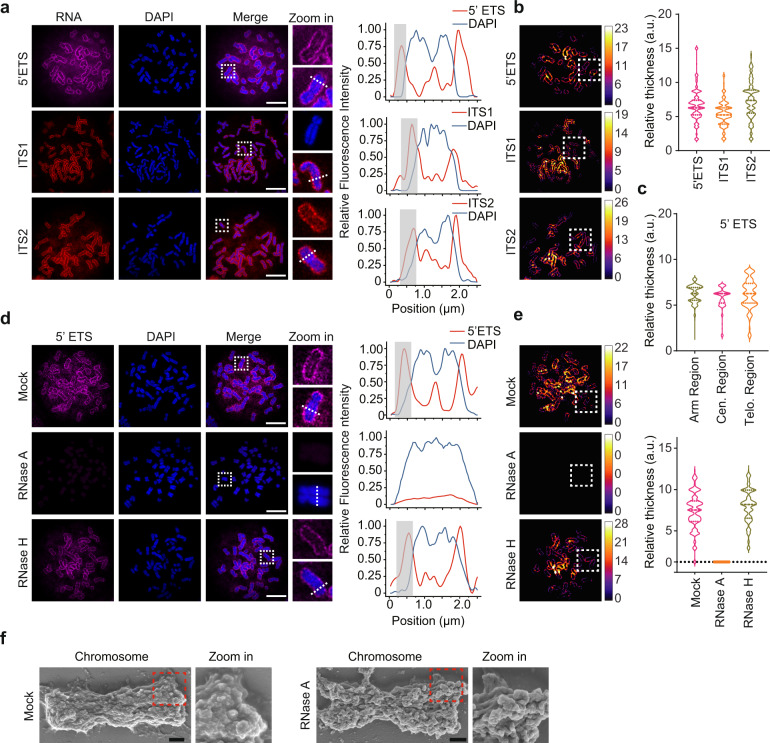
pre-rRNA localizes at the periphery of mitotic chromosomes. **a** Localization of 45S (5’ ETS), 30S (ITS1), and 32S (ITS2) pre-rRNAs at the perichromosomal layer. The RNA species were examined by RNA FISH with Cy5-labeled probe against 5’ETS, Cy3-labeled probes against ITS1 and ITS2 regions of pre-rRNAs in mitotic spreads. Chromosomes were counterstained with DAPI. Representative images are shown (left panel). The fluorescence signals on the white dash lines were examined (right panel), gray shadows indicate the perichromosomal layer of 45S, 30S, and 32S pre-rRNAs. Scale bar, 10 μm. **b** RNA is extended from the condensed chromosomes. The RNA FISH signal was analyzed by “Local Thickness” of Fiji to calculate the average RNA extension distance on the periphery of chromosomes, the dashed squares show the region of interest with isolated single chromosome. **c** The extension of 45S pre-rRNA on the periphery of different regions of each chromosome. **d** RNase A treatment abolishes the RNA species at the periphery. The mitotic spreads were treated with or without RNase A or RNase H. The fluorescence intensity of 45S rRNA was measured at the indicated section with white lines, gray shadow shows the perichromosomal layer of 45S pre-rRNA (right panel). Scale bar, 10 μm. **e** The RNA FISH signal was analyzed by “Local Thickness” of Fiji to calculate the average thickness of the 45S pre-rRNA coated at the periphery of chromosomes. Violin plots show the extension of RNA at the periphery. **f** SEM shows the detailed structure of chromosome treated with or without RNase A. Scale bar, 400 nm.

Next, we examined the localization of these rRNA species on the condensed chromosomes. With the probe targeting 5’ ETS, ITS1, or ITS2 region, we performed RNA FISH on metaphase spreads. Since these probes only recognize pre-rRNAs, we found that pre-rRNAs localized at the periphery of chromosomes (Fig. 2a). The extension of RNA on mitotic chromosomes was similar when mitotic spreads were examined with different probes (Fig. 2b). We also used other probes to examine 28S and 18S rRNAs and confirmed that chromosome-associated rRNA species exist at the periphery (Supplementary Fig. S2a). As the negative controls, ITS1 and ITS2 sense probes failed to detect any RNA species on the mitotic chromosomes (Supplementary Fig. S2b). To test if this is a universal phenomenon, we also examined mouse embryonic fibroblasts (MEFs) and found that all the rRNAs had similar localization pattern on mitotic chromosomes (Supplementary Fig. S3).

In addition, to further validate the specificity of rRNA on mitotic chromosomes, we also examined the GAPDH mRNA and NEAT1, an abundant noncoding RNA localized in nuclear paraspeckles. However, we didn’t observe these RNAs at the periphery of chromosomes. In contrast, using these probes, we could detect these RNAs in the interphase cells. In particular, NEAT1 probes stained nuclear paraspeckles clearly (Supplementary Fig. S4).

Moreover, we examined single chromosomes with 5’ ETS probes, and found that pre-rRNA covered every mitotic chromosome that we observed (Supplementary Fig. S5a). We also measured the coverage of these rRNAs on different regions of mitotic chromosomes and did not observe obvious differences among chromosome arms, centromeres, and telomeres (Fig. 2c). This result indicates that the rRNAs coat the periphery of chromosomes relatively evenly.

To further examine the status of these RNA species, we used RNase A or RNase H to treat mitotic spreads. Interestingly, RNase A but not RNase H treatment removed chromosome-associated rRNA species (Fig. 2d, e and Supplementary Fig. S5b, c). RNase A prefers to digest single-stranded RNA, whereas RNase H cleaves RNA:DNA hybrid. These results suggest that these rRNA species do not form RNA:DNA hybrid on mitotic chromosome. We also performed scanning electron microscopy (SEM) to examine mitotic chromosomes at high resolution. RNase A treatment caused the loss of materials associated with mitotic chromosomes, resulting in a bony morphology (Fig. 2f). Collectively, these results demonstrate that RNA species are major outer layer components of mitotic chromosomes.

### rRNA dynamically relocates onto mitotic chromosomes

**Fig. 3:**
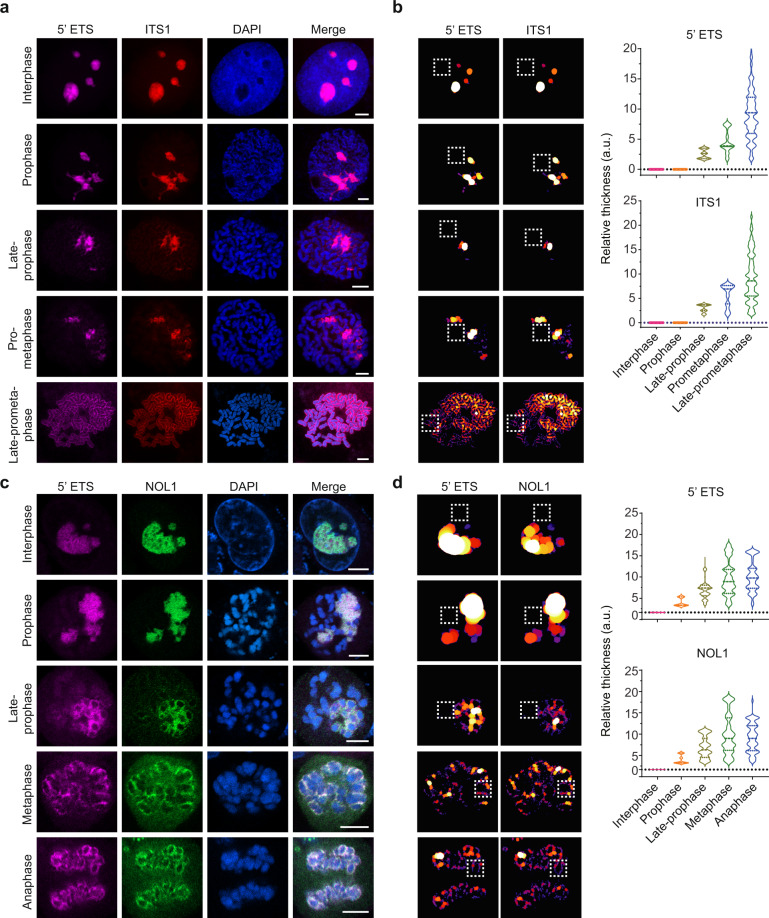
rRNA dynamically covers mitotic chromosomes. **a** The localization of pre-rRNAs from interphase to metaphase. pre-rRNAs were examined by RNA FISH in mitotic spreads. Scale bar, 5 μm. **b** The RNA FISH signals were analyzed by “Local Thickness” of Fiji. **c** The localization of pre-rRNAs and NOL1 from interphase to anaphase. 45S pre-rRNA was examined by RNA FISH, and NOL1 was detected by a fused EGFP tag. Scale bar, 5 μm. **d** The RNA FISH and EGFP signals were analyzed by “Local Thickness” of Fiji.

The pre-rRNA species mainly localize in the nucleolus during interphase. Along with nuclear envelope breakdown, the nucleolus is disassembled. We next examined if pre-rRNA associated with mitotic chromosomes in a dynamic process. Using mitotic spreads, we found that nucleoli were still buried in condensing chromosomes when the nuclear envelope started to breakdown. Along with chromosome condensation, nucleoli started to disassemble and pre-rRNAs gradually coated on mitotic chromosomes (Fig. 3a, b). Although mitotic spreads allow us to clearly observe the process of pre-rRNA coating onto mitotic chromosomes, we were unable to distinguish each mitotic phase with mitotic spreads. Thus, we performed RNA FISH on mitotic chromosomes for cells seeded on the coverslip. Interestingly, we found that rRNAs covered all mitotic chromosomes from prometaphase to anaphase (Fig. 3c, d). It is known that both nuclear envelope and nucleolus start to reassemble during cytokinesis^22,23^. Since mitotic chromosomes are decondensed during cytokinesis, it is difficult to clearly observe mitotic chromosomes at this stage.

The periphery of mitotic chromosomes consists not only rRNAs, but also a large number of proteins, many of which were relocated from nucleoli^17,24^. The best-characterized peripheral proteins include nucleolin (NCL), fibrillarin (FBL), and nucleophosmin (B23)^6,17,22^. We confirmed the localization of NCL and FBL at the periphery of chromosomes (Supplementary Fig. S6a). Moreover, GFP-FBL was expressed in the cells, and it clearly localized at the periphery (Supplementary Fig. S6b). In addition, NOL1, an rRNA methyltransferase identified in the nucleolus^10^, also localizes at the periphery of mitotic chromosomes (Supplementary Fig. S6b). We further examined the dynamic relocation of NOL1 to the periphery of chromosomes during mitosis. Similar to pre-rRNAs, NOL1 gradually translocated to the periphery of chromosomes during prometaphase, and remained associated with condensed chromosomes till anaphase (Fig. 3c, d). In contrast, we found that mature ribosome protein subunits did not associate with chromosomes (Supplementary Figs. S7 and S8), indicating that it is not the mature ribosome that associates with mitotic chromosomes. In addition to protein partners, other RNA species, such as snoRNAs, also associate with pre-rRNAs^7,17,18^. Thus, we also examined snoRNAs and found that snoRNAs, such as U3, U8, and U17, localized at the periphery of mitotic chromosomes (Supplementary Fig. S9).

### Ki-67 mediates the association of pre-rRNAs with chromosomes

**Fig. 4:**
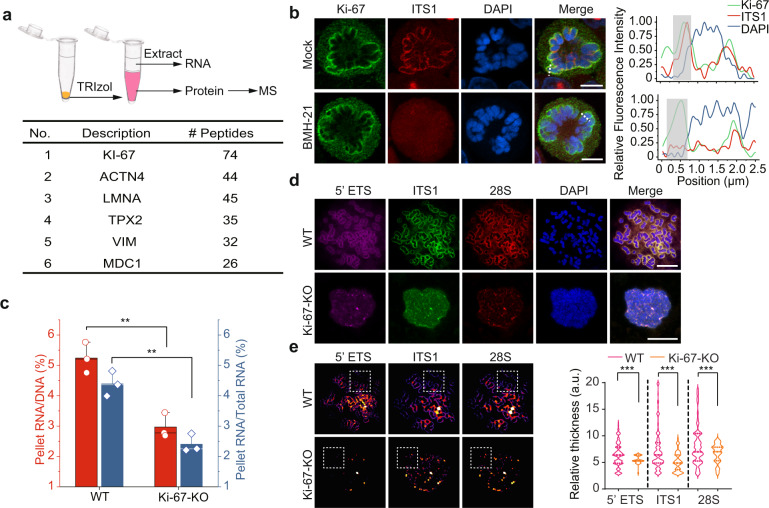
Ki-67 mediates pre-rRNA’s association with chromosomes. **a** The protein species in the pellet fraction. The protein species was isolated from TRIzol extraction and analyzed by mass spectrometry. The top six abundant proteins are shown. **b** The localization of Ki-67 and pre-rRNA at the periphery of chromosomes. Pre-rRNA was examined by RNA FISH with probes against the ITS1 region in mitotic cells treated with or without BMH-21. Ki-67 was examined with anti-Ki-67 antibody. Signal intensity of pre-rRNA, Ki-67, and DAPI at the indicated section was analyzed (right panel). Scale bar, 5 μm. **c** The ratio of RNA in the pellets to total DNA mass (left *y* axis) and the ratio of RNA in the pellets to total RNA (right *y* axis) in parental 293T and Ki-67 knockout cells. **P < 0.01. **d** Localization of pre-rRNAs detected by RNA FISH with Cy5-labeled probe against 5’ETS, FAM-labeled probes against ITS1 and Cy3-labeled probe against 28S region of pre-rRNAs at the perichromosomal layer in parental 293T and Ki-67-knockout cells. Scale bar, 10 μm. **e** The RNA FISH signals were analyzed by “Local Thickness” of Fiji, the dashed square shows the region of interest. ***P < 0.001.

The fact that RNase H could not remove pre-rRNAs from condensed chromosomes indicates that RNA:DNA hybrids are not formed. It further suggests that DNA itself may not be sufficient to retain pre-rRNAs on chromosomes. We hypothesized that protein species may be required for the localization of pre-rRNAs. To identify the possible protein species, we examined protein components in the condensed chromosome fraction with mass spectrometry. Interestingly, the top candidate is Ki-67 (Fig. 4a and Supplementary Table S1), a nuclear protein known to associate with pre-rRNAs as well as localize at the periphery of mitotic chromosomes^8,13,16^.

We arrested 293T cells at G2/M phase with RO-3306, a CDK1 inhibitor for overnight (Supplementary Fig. S10), followed by the treatment with or without RNA polymerase I inhibitor BMH-21 for 4 h to abolish the transcription of pre-rRNAs. The cells were then released into mitosis and arrested at prometaphase in the presence of colcemid. Since the pre-rRNAs have been depleted by the treatment of BMH-21, we did not observe pre-rRNA species at the periphery of chromosomes (Fig. 4b). Along with the loss of pre-rRNAs, NOL1 was no longer covered on mitotic chromosomes (Supplementary Fig. S11), suggesting that NOL1 is tethered to chromosomes by pre-rRNAs. In sharp contrast to NOL1, Ki-67 still localized at the periphery of mitotic chromosomes (Fig. 4b), suggesting that the localization of Ki-67 at the peripheral layer is not dependent on pre-rRNAs.

Next, we generated Ki-67-deficient cells using CRISPR/cas9 system. The Ki-67-knockout cells were verified by genomic sequencing and western blotting (Supplementary Fig. S12a, b). The rRNA transcription and processing were not obviously altered in the Ki-67-deficient cells (Supplementary Fig. S12c), and the morphology of nucleoli in the interphase of Ki-67-deficient cells was unchanged (Supplementary Fig. S12d). Surprisingly, chromosome-associated RNAs were largely reduced in the Ki-67-deficient cells (Fig. 4c). Very small amounts of rRNA species, including 45S, 30S, and 28S rRNAs, attached onto the chromosomes (Fig. 4d, e and Supplementary Fig. S12d). In addition, the chromosomes at the metaphase of the Ki-67-deficient cells collapsed into a chromatin mass (Fig. 4d, e and Supplementary Fig. S12d), which is consistent with the previous studies^16^.

**Fig. 5:**
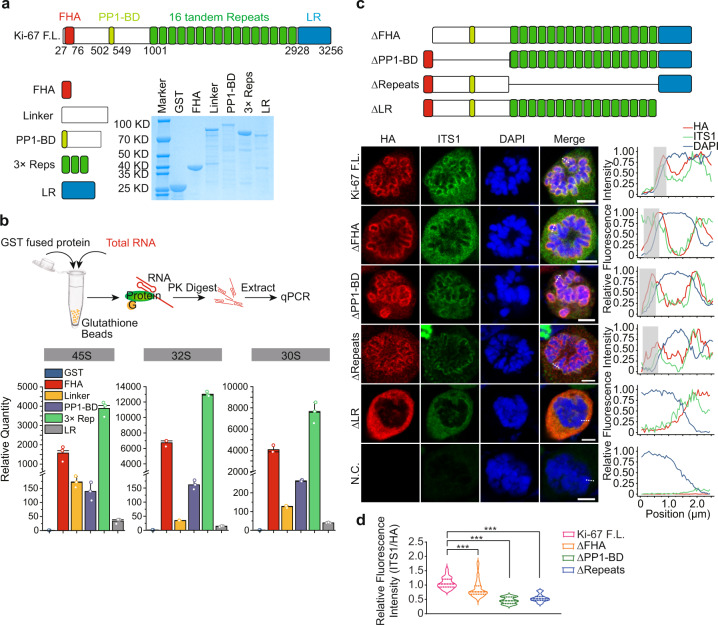
Mapping the domains of Ki-67 interacting with pre-rRNAs. **a** A schematic diagram of each domain of Ki-67. Recombinant proteins of each domain were examined by Coomassie brilliant blue (CBB). **b** Ki-67 associates with rRNA. Upper panel shows the workflow of protein pulling down assays. Recombinant proteins were incubated with total RNA. The RNA pull-down assays were performed followed by RT-qPCR analyses. The location of primers is shown in Supplementary Fig. S1e. **c** Ki-67 plays an important role in chromosome dispersion. Upper panel shows a schematic diagram of internal deletion mutants of Ki-67. Lower left panel shows the chromosome dispersion in the Ki-67-deficient cells reconstituted with full-length and mutant Ki-67. RNA FISH was detected by Cy3-labeled probe against ITS1 region, HA-tagged full-length, and truncations of Ki-67 were detected by anti-HA antibody. Lower right panel is the line analysis of the pre-rRNA, Ki-67, and DAPI signal intensity at the indicated section. Scale bar, 5 μm. **d** Statistic analysis of the relative fluorescence intensity of ITS1 probe along the chromosome. The results were normalized by Ki-67 expression (the signal intensity of HA tag). ***P < 0.001.

Ki-67 is a large polypeptide with 3256 residues, which contains four prominent regions, namely an N-terminal forkhead-asssociated (FHA) domain^25^, a protein phosphatase 1-binding domain (PP1-BD)^13^, a large disordered region with 16 tandem repeats^26^, and a C-terminal LR domain^27^ that recognizes genomic DNA^16^. We generated recombinant proteins containing each domain and examined the interactions between these domains and rRNAs (Fig. 5a). Interestingly, we found that the FHA domain, PP1-BD and the repeat region were associated with rRNAs (Fig. 5b). Because 16 tandem repeats of Ki-67 are too large and difficult to be expressed, we only generated a recombinant protein with three tandem repeats. Even this truncated repeat domain could efficiently enrich pre-rRNAs, as compared to the control (Fig. 5b). However, 18S, 5.8S, and 28S rRNAs were only modestly enriched (Supplementary Fig. S13).

Of note, the FHA domain is known as a phospho-Thr-binding motif^25^. Although the putative phosphate-binding pocket has been mapped in Ki-67 FHA domain, protein binding partners have not been identified yet. We mutated the key residues (R31A and K45A) in the putative phosphate-binding pocket of the FHA domain and found these mutations abolished the interaction with rRNAs (Supplementary Fig. S14), suggesting that the FHA of Ki-67 is a bona fide nucleic acid-binding motif.

Next, we generated internal deletion mutations to abolish each prominent domain (Fig. 5c). We expressed full-length and mutants of Ki-67 in the Ki-67-deficient cells. The full-length of Ki-67 was able to completely restore pre-rRNAs at the periphery of chromosomes (Fig. 5c). Although the mutants without the FHA, PP1-BD or repeats domain could partially restore pre-rRNAs at the periphery of chromosomes, the signal intensity of pre-rRNAs on the chromosomes was clearly reduced (Fig. 5c, d). Moreover, the R31A or K45A mutants impaired the loading of rRNA at the periphery of mitotic chromosomes (Supplemental Fig. S15a, b). In addition, the LR domain deletion mutant cannot restore the single chromosome status probably due to its loss of DNA binding (Fig. 5c). Collectively, these results indicate that the FHA domain, PP1-BD, and the repeat region recognize pre-rRNAs, whereas the LR domain binds DNA and anchors Ki-67 onto mitotic chromosomes (Fig. 5c, d).

### Pre-rRNAs facilitate chromosome dispersion at prometaphase

**Fig. 6:**
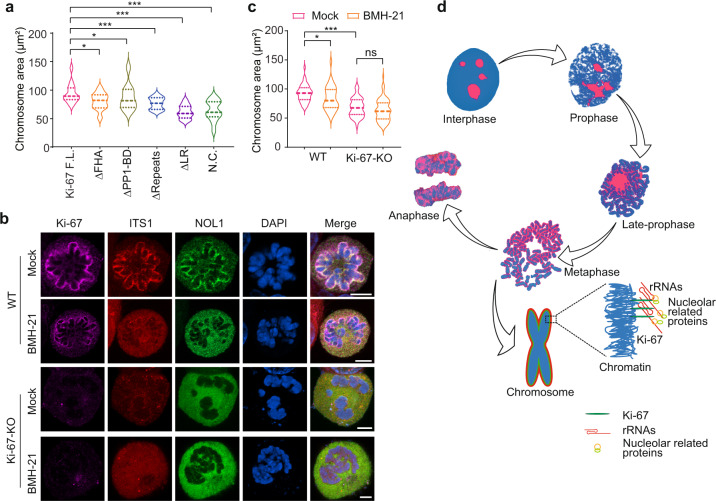
Pre-rRNA facilitates chromosome dispersion at prometaphase. **a** Ki-67 promotes chromosome dispersion. Chromosome areas at prometaphase were measured in the cells expressing full-length Ki-67 or indicated Ki-67 mutants. **b** RNA polymerase I inhibitor treatment suppresses chromosome dispersion. Parental 293T cells or Ki-67-deficient cells were treated with BMH-21. The localization of Ki-67, pre-rRNA, and NOL1 was examined. Scale bar, 5 μm. **c** Chromosome areas were analyzed by ImageJ. *P < 0.05; **P < 0.01; ***P < 0.001. **d** A schematic model shows that pre-rRNAs gradually translocated from nucleoli to the periphery of chromosomes during mitosis. Ki-67 anchors these rRNA species on the condensed chromosomes.

Loss of Ki-67 impairs mitotic chromosome dispersion from late prometaphase to metaphase^16^. We also found that the chromosomes in Ki-67-deficient cells were indeed clustered together during metaphase (Fig. 4d). We measured the chromosome area^16^, and found that loss of Ki-67 reduced the overall chromosome area of metaphase cells, and this defect was rescued by the full-length Ki-67 (Fig. 6a). Consistently, reconstitution of the Ki-67-deficient cells with those internal deletion mutants, including ΔFHA, ΔPP1-BD, ΔRepeats and the R31A and K46A mutants only slightly increased the chromosome area (Fig. 6a and Supplementary Fig. S15). Since these domains mediate the interaction with rRNAs, these results suggest that rRNA binding may contribute to the dispersion of condensed chromosomes during metaphase.

Next, we suppressed pre-rRNA transcription by transiently treating G2/M phase cells with the RNA polymerase I inhibitor. After releasing the cells from G2/M phase, we immediately added colcemid to arrest cells at prometaphase. As expected, rRNA species were not observed on mitotic chromosomes. And importantly, the loss of rRNAs reduced chromosome areas (Fig. 6b, c). Moreover, suppression of pre-rRNA biogenesis did not further reduce chromosome area in Ki-67-deficient cells (Fig. 6a, b). Thus, this epistasis effect indicates that Ki-67 and pre-rRNAs may act in the same pathway to disperse chromosomes in metaphase.

## Discussion

In this study, we have provided the first evidence that rRNAs are major chromosome-associated RNA species and cover whole chromosomes. We found that pre-rRNA was highly enriched at the periphery of mitotic chromosomes. Meanwhile, we also detected 18S, 5.8S, and 28S rRNAs on the mitotic chromosomes. During pre-rRNA processing, 18S, 5.8S, and 28S rRNAs also exist in nucleoli. Thus, it is possible that these rRNA species on mitotic chromosomes are still from disassembled nucleoli. Of note, we did not observe 5.8S rRNA in the soluble fraction during RNA sequencing. It might be because 5.8S rRNA only has 156 nt, and is much smaller than 18S and 28S rRNAs. During the RNA sequencing library preparation, 5.8S rRNA fragments were lost. Thus, we did not observe abundant 5.8S rRNA in RNA sequencing results in the soluble fraction. In contrast, in the pellets, we harvested a large amount of pre-rRNA that includes the sequence of 5.8S region. Some of the sequencing reads of pre-rRNA were mapped at the 5.8S region, and were shown in Fig. 1c. Nevertheless, we clearly detected 5.8S rRNA in soluble fractions using northern blotting (Fig. 1d).

In addition to rRNAs, other rRNA-associated RNAs, such as snoRNAs, also bind to mitotic chromosomes. Via 5’-tag deep sequencing, earlier studies identified that numerous noncoding RNA species associate with chromosomes^28^. Interestingly, more than 50% of noncoding RNA reads were mapped on snoRNA clusters^28^. However, compared to the abundance of rRNAs, these RNA species are unlikely the major RNA species associated with mitotic chromosomes. Since snoRNA is associated with pre-rRNA for rRNA maturation, it is possible that these snoRNAs and a set of pre-rRNA-associated proteins, such as FBL, NCL, and NOL1, act as passengers to coat mitotic chromosomes. Moreover, we did not find mature ribosomes or mRNAs associating with mitotic chromosomes (Supplementary Figs. S4a and S7). Thus, we exclude the possibility that the role of rRNAs on mitotic chromosomes is for protein translation.

Previous studies have indicated that rRNAs may associate with condensed chromosomes during mitosis^29–31^. However, the localization of rRNAs on mitotic chromosome has not been systematically examined. In particular, the mechanism underlying the recruitment of rRNAs to the condensed chromosomes is unclear. Here, we show that Ki-67 plays a key role to mediate the relocation of pre-rRNAs to mitotic chromosomes. Similar to Ki-67, pre-rRNAs are evenly distributed to the chromosome periphery. Ki-67 interacts with pre-rRNAs via multivalent interactions involving the FHA domain, PP1-BD, and the Ki-67 repeats (Fig. 5b). The FHA domain is known to bind phospho-threonine^32^. However, the binding partner of Ki-67 FHA domain has not been identified until our study. It is likely that the FHA domain of Ki-67 recognizes the phosphate group in the rRNA backbone. The key residues (R31A and K45A) mutation in the phosphate-binding pocket of the FHA domain abolished the interaction with rRNA. This is also the first evidence to demonstrate that the FHA is a nucleic acid-binding motif. Additional structure analysis of the complex of Ki-67 and rRNAs is needed to further define this interaction in detail. Nevertheless, it is clear that Ki-67 anchors pre-rRNAs to mitotic chromosomes. The LR domain of Ki-67 recognizes the genomic DNA, whereas the FHA domain, PP1-BD, and the Ki-67 repeats bind RNA. Ki-67 acts as a bridge to connect pre-rRNAs to condensed chromosomes (Fig. 6d). However, the genetic ablation of Ki-67 cannot completely remove all chromosome-associated pre-rRNAs. It is possible that other protein partners may also mediate the interaction between rRNAs and mitotic chromosomes. In addition, ribosomes are still normal assembled in Ki-67-deficient cells, transcription and processing of pre-rRNA are not impaired in Ki-67-deficient cells^14^ (Supplementary Fig. S12c). Although Ki-67 localizes in nucleoli in the interphase, it is possible that other factors may play redundant roles for rRNA biogenesis in the absence of Ki-67.

It has been shown that Ki-67 mediates chromosome dispersion during late prometaphase to metaphase. The current paradigm posits that, as a highly charged polypeptide, Ki-67 mediates chromosome dispersion by acting as a surfactant and via charge repulsion. However, Ki-67 is a lysine- and arginine-rich polypeptide with an overall positive charge. Because DNA is negatively charged, Ki-67 is unlikely to induce chromosome dispersion via simple charge-charge repulsion. In this study, we found that rRNAs promoted chromosome dispersion as well. Since rRNAs are negatively charged, it is possible that Ki-67 brings rRNA species to chromosomes, which in turn induces chromosome dispersion. Our results support this hypothesis, as suppression of rRNA biogenesis impairs chromosome dispersion.

The rRNA coating on chromosomes may also have additional functions during mitosis as rRNAs localize at the chromosome periphery like finger gloves. It may protect the genomic DNA during mitosis so that they can endure drastic microenvironmental changes during mitosis. Future studies may reveal additional functions of pre-rRNAs on mitotic chromosomes during different mitotic stages. Regardless, our findings demonstrate that rRNAs are major chromosome-associated RNA species, which play an important role in chromosome dispersion.

## Materials and methods

### Plasmids

Human full-length Ki-67 was subcloned from IRESpuro2-Ki-67, a gift from Dr. Daniel W. Gerlich, into the SBP vector. The SBP tag was then replaced by an HA tag. Truncation mutants were generated by site-directed mutagenesis. For ΔFHA, amino acids 1–100 were deleted; for ΔPP1-BD, amino acids 101–990 were deleted; for ΔRepeats, amino acids 995–2945 were deleted; for ΔLR, amino acids 2931–3256 were deleted; FHA: amino acids 1–100; Linker: amino acids 101-512; PP1-BD, amino acids 512–987; 3× repeats: amino acids 988–1363; LR: amino acids 2931–3256. Human full-length NOL1 was cloned into the SBP vector and replaced the SBP tag with a GFP tag.

### Cell culture and transfection

293T and HCT116 cells were cultured in DMEM containing 10% fetal bovine serum and supplemented with 100 U/mL penicillin and 100 μg/mL streptomycin at 37 °C with 5% CO_2_ (v/v). Transfection was performed in 70%–80% confluent cells using Lipofectamine^®^ 2000 according to the manufacturer’s instructions. Ki-67 knockout cell lines were established by CRISPR/cas9 system. The annealed sgRNA oligos were cloned into pSpCas9(BB)-2A-Puro, and transfected in 293T cells to select stable cell lines by puromycin treatment.

### Extraction of Chromosome-associated RNAs

HCT116 cells were arrested by colcemid at metaphase, then collected and lysed by NETN100 buffer (0.5% NP-40, 50 mM Tris-HCl, pH 8.0, 2 mM EDTA, and 100 mM NaCl). After centrifugation, the pellet was washed at least three times with NETN100 buffer, finally RNA in the pellet was extracted by TRIzol reagent (Life Technologies) according to the manufacturer’s protocol and analyzed by northern blotting and/or qPCR, as described below.

### RNA electrophoresis

Before loading onto the agarose gel, the RNA was mixed with 18% formaldehyde in 1× MOPS buffer, denatured for 10 min at 65 °C. For analysis of the chromosome-associated rRNA species, 1 μg of total RNA was loaded onto the agarose denaturing gel (10% formaldehyde/1% agarose in MOPS buffer).

### RNA-seq library preparation and sequencing

Library preparation and sequencing were finished by BGI company (Shenzhen, China). The protocol is as follows: (1) RNA fragment: RNA molecules were fragmented into small pieces using fragmentation reagent; (2) cDNA synthesis: first-strand cDNA was generated using random hexamer-primed reverse transcription, followed by a second-strand cDNA synthesis; (3) End Repair and Adaptor Ligation: the synthesized cDNA was subjected to end-repair and then was 3’ adenylated. Adapters were ligated to the ends of these 3’ adenylated cDNA fragments; (4) PCR: this process was to amplify the cDNA fragments with adapters from the previous step. PCR products were purified with Ampure XP Beads (AGENCOURT), and dissolved in EB solution; (5) Library Quality Control: library was validated on the Agilent Technologies 2100 bioanalyzer; (6) Circularization: The double-stranded PCR products were heat-denatured and circularized by the splint oligo sequence. The single-strand circle DNA (ssCir DNA) was formatted as the final library; (7) Sequencing: the library was amplified with phi29 to make DNA nanoball (DNB) which had more than 300 copies of one molecular. The DNBs were loaded into the patterned nanoarray and single end 50 (pair-end 100/150) bases reads were generated in the way of combinatorial Probe-Anchor Synthesis (cPAS).

### Bulk RNA-Seq analysis

The human 45S rDNA locus reference sequences and gff annotation file (GFF3 format) were obtained from GenBank (Accession No. U13369.1). The human genome reference (GRCh38.p13) and annotation file were downloaded from GENCODE.

The raw fastq files were analyzed using FastQC (v0.11.9). Sequencing adaptors and low quality (base quality score < 20) reads were trimmed by trim galore (v0.6.6). Reads shorter than 20 nt were discarded. The trimmed reads were aligned to the 45S rDNA locus sequences by STAR (v2.7.8a). The 45S rDNA annotation file was transformed to GTF format using gffread (v0.12.1). The expression levels of 18S, 5.8S, 28S rRNA, ITS1, ITS2, 5’ETS, 3’ETS were quantified by FeatureCounts (v2.0.1). The normalized counts (counts per million, CPM) in 18S rRNA, 28S rRNA, ITS1, ITS2, 5’ETS, 3’ETS regions were quantified in each 100-bp bin, and the normalized counts in 5.8S rRNA region were quantified in each 10-bp bin (Fig. 1c). In addition, the reads that were not mapped to 45S rDNA were aligned to the human GRCh38 genome and quantified by FeatureCounts (v2.0.1). The rRNA biotype fraction were calculated by the “biotype” annotation.

### Northern blot

Agarose gels were transferred by capillarity overnight in 20× saline sodium citrate (SSC). Membranes were pre-hybridized for 1 h at 42 °C in ULTRAhyb™ Ultrasensitive Hybridization Buffer (Invitrogen), the biotin-labeled probe was added and incubated for overnight at 42 °C. Sequences of the probes are described in Supplementary Table S2. After hybridization, membranes were washed three times with 0.2× SSC/0.1% SDS for 15 min at 42 °C. The biotin signal was finally detected by Chemiluminescent Nucleic Acid Detection Module Kit (Thermo Scientific) according to the manufacturer’s protocol.

### Quantitative real-time PCR

Total RNA was extracted with TRIzol Reagent (Invitrogen) according to the manufacturer’s protocol. cDNA was generated with Superscript III reverse transcriptase (Invitrogen). Quantitative PCR was performed with triplicate samplings of retrotranscribed cDNAs on a Bio-Rad Real-Time PCR System with SYBR Green PCR core reagents (Bio-Rad). Primers for qPCR reactions are summarized in Supplementary Table S2. The mean value was calculated by three independent experiments. Since pre-rRNAs also contain 18S, 5.8S, and 28S region. When calculating the enrichment of these mature rRNA species in the pull-down assays, we subtracted the pre-rRNA during the data analysis.

### Mitotic chromosome spreads

Cells were trypsinized and resuspended in 75 mM KCl for 10 min at 37 °C. Cells were then fixed by 3:1 ice-cold methanol:acetic acid for 15 min at room temperature. After two washes with 3:1 ice-cold methanol:acetic acid, cells were dropped on cleaned and pre-chilled glass slides from a height of 30 cm. Cells were dried on the slide and ready for following experiments. Line scan analyses of pre-rRNAs signal were done on chromosome spreads by ImageJ. The RNA extension signal was analyzed by Fiji-”Local Thickness”, which transforms fluorescence signals into the distance map that is shown in the violin plots.

### Scanning electron microscopy analysis

Scanning electron microscopy analysis of chromosome was performed according to the drop/cryo technique^33^. 293T cells were pretreated with colcemid for overnight to be arrested at metaphase, then cells were collected and treated with hypotonic buffer (75 mM KCl), subsequently fixed in 3:1 ethanol: acetic acid. One drop of chromosome suspension was placed on a glass slide and covered by a coverslip. Coverslips were removed after freezing on solid CO_2_. The slides were immediately immersed in 2.5% glutardialdehyde fixative buffer (50 mM cacodylate, 2 mM MgCl_2_, pH 7.2) and washed three times in buffer. The slides were then dehydrated through a graded ethanol series (20%–100%), air dried using hexamethyl disilylamine (HMDS), and sputtered with 3 nm of gold/palladium. Chromosomes were then viewed with Gemini SEM Crosssbeam 550.

### Inhibitors and stains

Colcemid (Roche) was used at a final concentration of 100 ng/mL for overnight to arrest cells at prometaphase. CDK1 inhibitor RO-3306 was used at a final concentration of 10 μM for overnight to arrest cells in G2/M phase. For analyzing the dynamic process of nucleolar changes, cells were treated with CDK1 inhibitor overnight, then wells were washed three times with 1 ml fresh medium to release cells into cell cycle. For RNA polymerase I inhibitor-treated samples, BMH-21 were added 4 h before RO-3306 washout. Following the treatment, cells were released into fresh media for 30 min and colcemid was added to arrest cells at prometaphase. Then cells were prepared for following RNA FISH or immunostaining. The mitotic chromosome areas were unbiasedly examined from 20 to 30 prometaphase cells that were randomly selected under the same stage of mitosis. The statistics were analyzed and shown in violin plot to reflect mean value and interquartile range.

### RNA FISH

The RNA FISH probes are summarized in Supplementary Table S3. The samples were hybridized with the different regions of 45S RNA FISH Probe set labeled with Cy3 or Cy5, following the manufacturer’s instructions available online at www.biosearchtech.com/stellarisprotocols. Briefly, adherent cells were fixed in 3% paraformaldehyde for 15 min at room temperature and then permeabilized with 1 mL of 70% (v/v) ethanol for at least 1 h at 2–8 °C. Mitotic spread chromosome samples were directly treated with 70% ethanol. Then aspirate the 70% ethanol off the coverglass with washer buffer A for 5 min at room temperature. Incubate the samples with 100 μL of the hybridization buffer containing probes in the dark at 37 °C for 4–16 h, followed by wash buffer A incubate at 37 °C for 30 min. Aspirate the wash buffer A, and then add DAPI nuclear stain to counterstain the nuclei followed by mounting with anti-fade solution.

### Antibodies

The following antibodies were purchased from respective companies: anti-Ki-67 (Thermo Fisher, MA5-14520), anti-RPL3 (Proteintech, 11005-1-AP), anti-GAPDH (CST, 5174), anti-H2B (CST, 12364), anti-H4 (CST, 13919), anti-FBL (CST, 2639S), anti-NCL (CST, 14574), anti-RPS3(ABclonal, A2533), anti-RPL6 (Proteintech, 15387-1-AP), anti-RPL7A (Abclonal, A13713), anti-RPL8 (Proteintech, 16981-1-AP), anti-RPL10 (Proteintech, 17013-1-AP), anti-RPL13 (Proteintech, 11271-1-AP), anti-RPL14 (Proteintech, 14991-1-AP), RPL39 (Proteintech, 14990-1-AP), anti-HA-tag (MBL, M180-3).

### Immunostaining

Cells were fixed in 3% paraformaldehyde for 15 min at room temperature, washed in PBS, and permeabilized with 0.5% Trixton X-100 in phosphate-buffered saline (PBS) for 10 min at room temperature. Samples were blocked with 5% BSA and then incubated with the primary antibody for 1 h. Samples were washed three times and incubated with the secondary antibody for 30 min. After PBS wash, the nuclei were stained by DAPI. The coverslips were mounted onto glass slides and visualized with Zeiss LSM 880 with Airyscan. All the images were acquired with Zen software under Zeiss LSM 880 with Airyscan equipped with an 63×/1.4 oil immersion objective at room temperature. Contrast and brightness settings were identically performed on all images in a given experiment.

### Western blot

Cells were lysed with NETN100 buffer (0.5% NP-40, 50 mM Tris-HCl, pH 8.0, 2 mM EDTA, and 100 mM NaCl). Extracted proteins were separated by SDS-PAGE and transferred to PVDF membranes. After being blocked with TBST buffer containing 5% nonfat milk for 1 h at room temperature, the membranes were incubated 1 h at 37 °C in the primary antibodies. After being washed with TBST buffer, membranes were then incubated with secondary antibodies for 1 h at room temperature. Signals were detected using ECL western blotting substrate (Thermo Fisher) according to the manufacturer’s instructions.

### Statistical analysis

All data with error bars are presented as means ± SD. The significance of differences between treatment and control mean values in all the experiments was determined by two-tailed Student’s *t* test. ns, non-significant; *P < 0.05; **P < 0.01; ***P < 0.001. All calculations except the violin plot were performed using OriginPro software, and violin plots were performed using GraphPad Prism software.

## Supplementary information

Supplementary information

Supplementary Table 1

## Data Availability

All datasets have been deposited in the GEO Datasets under the GEO accession number GSE182987.
