# Supplementary material for: Ribosomal RNA regulates chromosome clustering during mitosis
Source: Cell Discov. 2022 May 31;8:51. doi: 10.1038/s41421-022-00400-7 (PMC9151767; doi:10.1038/s41421-022-00400-7)
Supplement: Supplementary file 1 — Supplementary information [file 41421_2022_400_MOESM1_ESM.pdf]

## Supplementary Figure

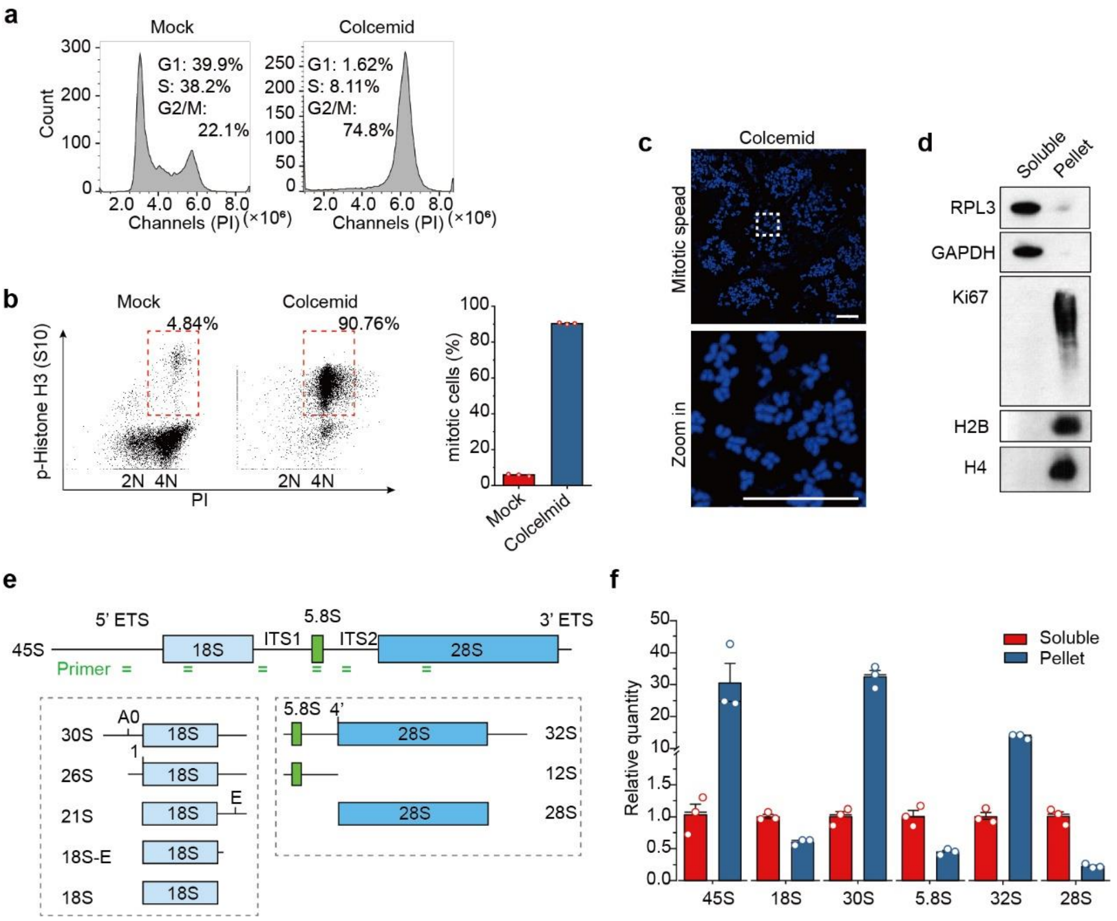


**Supplementary Figure S1. rRNA associates with chromosomes during mitosis. a**, The cell cycle of colcemid treated cells was examined by flow cytometry. **b**, The staining with propidium iodide (PI) and anti-histone H3pSer10 in mock and colcemid treated cells was analyzed by flow cytometry. The percentage of H3pSer10 cells from flow cytometry analysis is shown. **c**, Mitotic spreads show the separated condensed chromosomes. Interphase cells were not observed. Scale bar: 20μm. **d**, The soluble and pellet fractions isolated from colcemid treated cells was examined by western blotting with indicated antibodies. **e**, A schematic diagram shows the processing of pre-rRNAs. The position of probes for northern blot and primers for qPCR is shown. **f**, RNA species from soluble and pellet fraction were examined by qPCR.

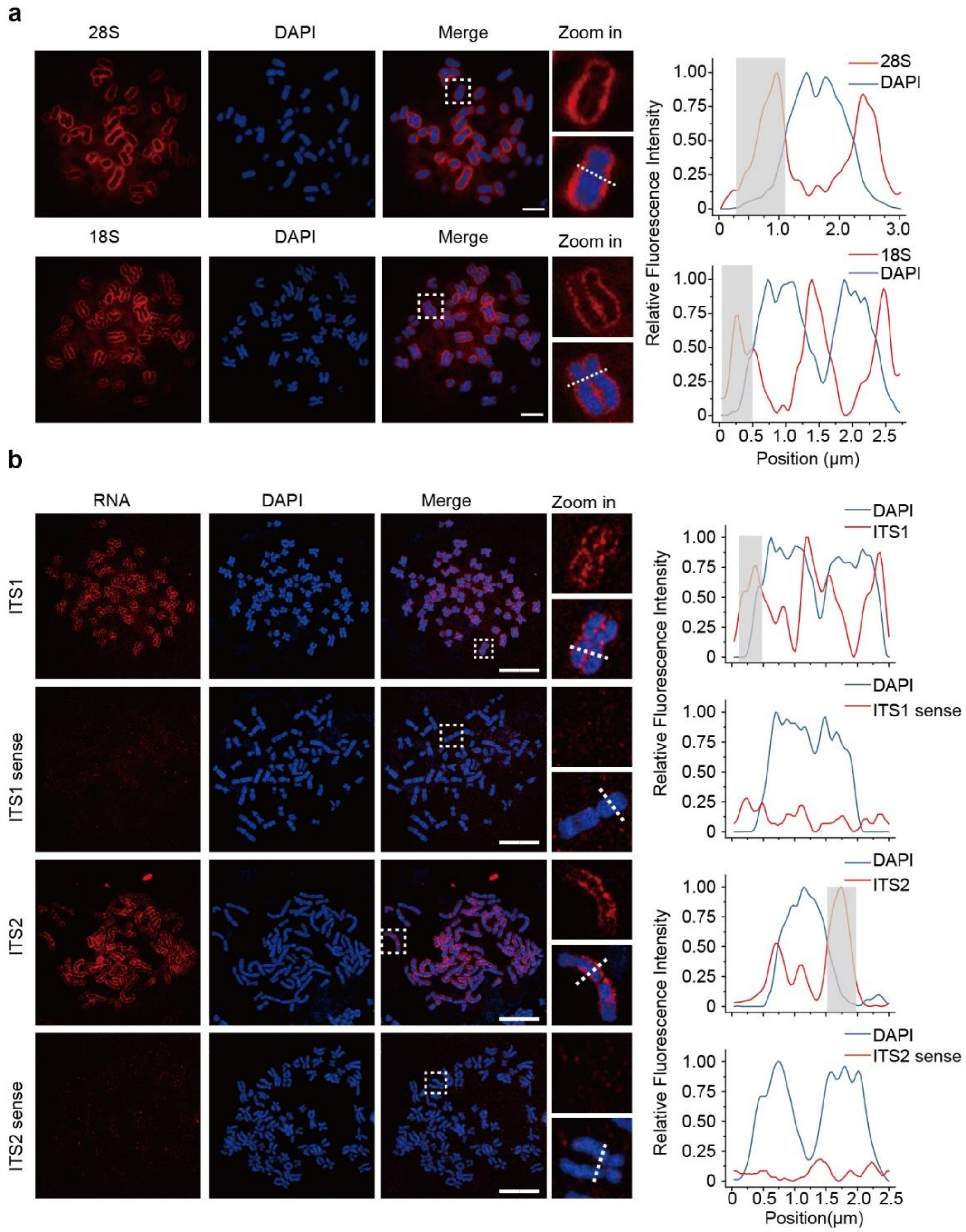


**Supplementary Figure S2. rRNA localizes at periphery of mitotic chromosomes. a**, RNA FISH was performed using Cy3-labeled probes against 28S or 18S region to examine rRNA on chromosomes. **b**, RNA FISH was performed using Cy3-labeled probes against ITS1 or ITS2 region. Sense ITS1 or ITS2 probes were used as negative control. Chromosomes were counter stained with DAPI. Representative images are shown in the left panel. The relative signal intensity of RNA and DAPI on the white

line of the indicated section was measured, grey shadow indicates the perichromosomal layer of pre-rRNAs, which is shown in the right panel. Scale bar: 5μm.

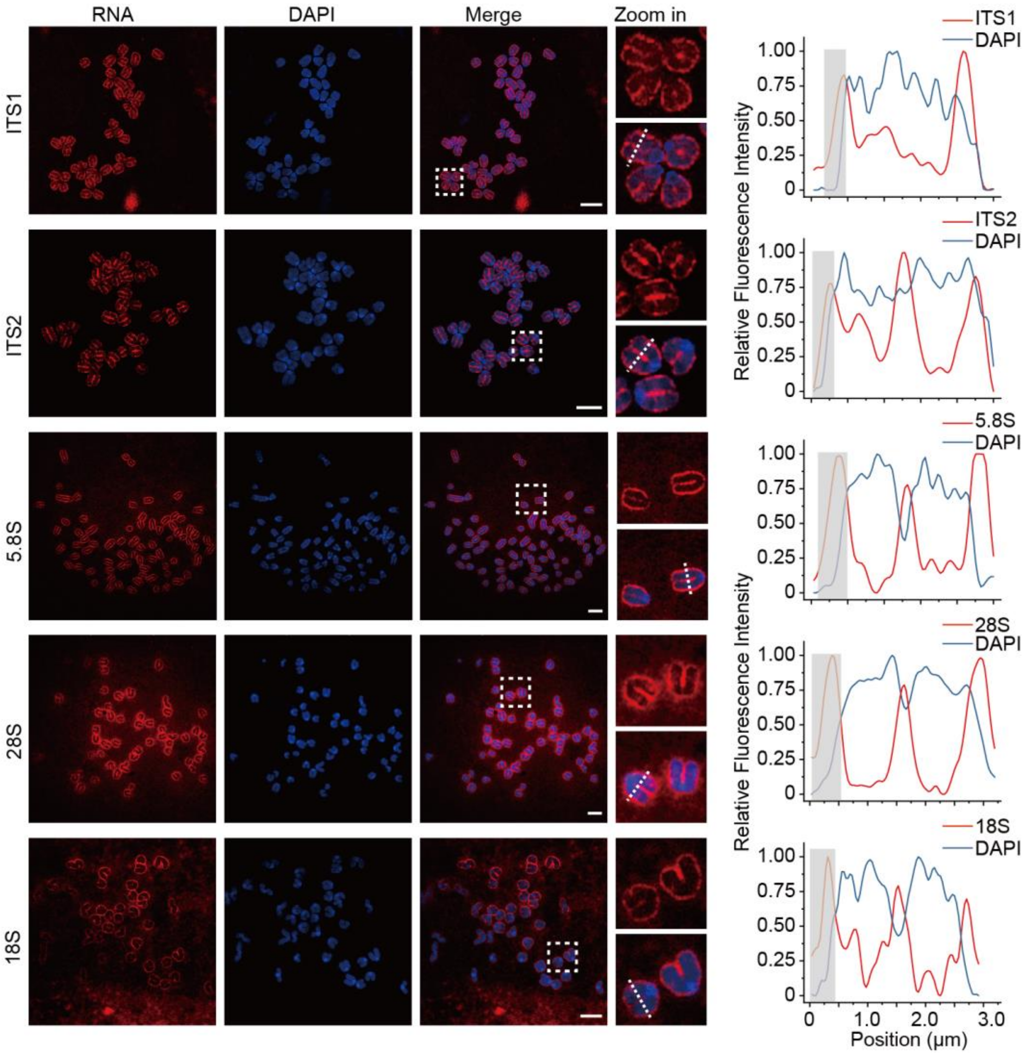


**Supplementary Figure S3. Pre-rRNA localizes at periphery of mitotic chromosomes in MEFs.** RNA FISH was performed using Cy3-labeled probes targeting ITS1, ITS2, 5.8S, 28S and 18S regions on mitotic spreads of MEFs. The relative signal intensity of RNA and DAPI on the white line of the indicated section was measured, grey shadow shows the perichromosomal layer of pre-rRNAs (right panel). Scale bar: 5μm.

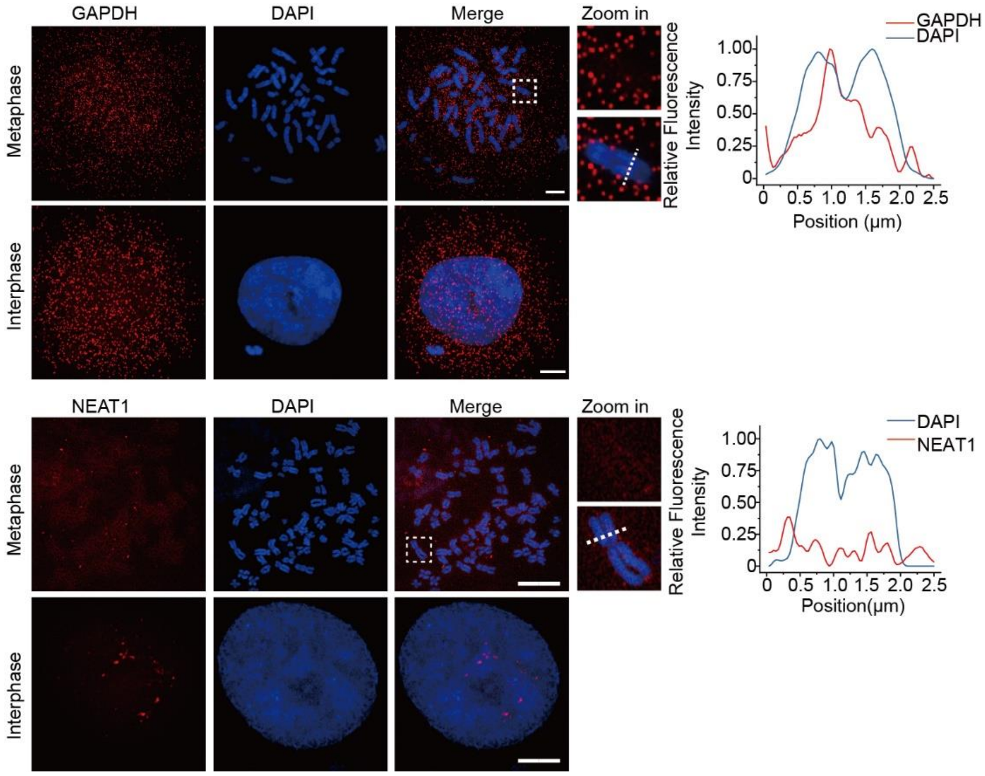


**Supplementary Figure S4. The localization of GAPDH and NEAT1 on chromosomes and interphase cells.** RNA FISH was performed using Cy3-labeled probes against GAPDH mRNA or NEAT1 noncoding RNA. The relative signal intensity of RNA and DAPI on the white line of the indicated section was measured, (right panel). Scale bar: 5μm.

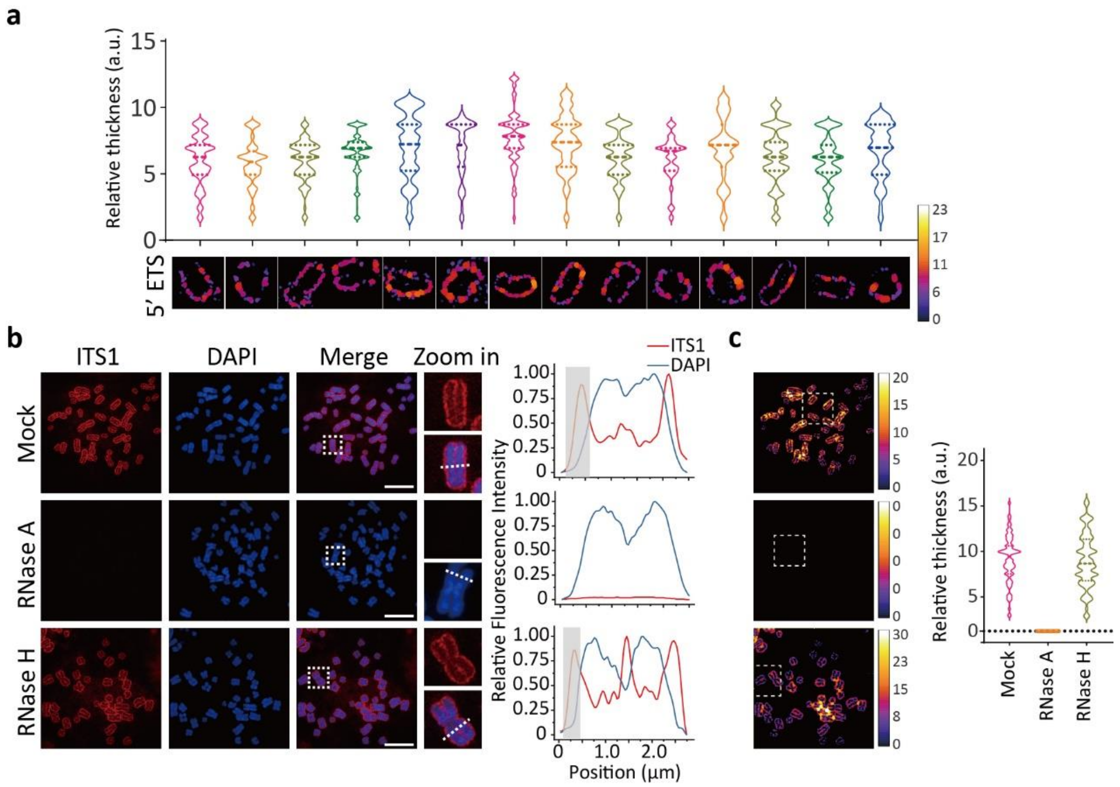


**Supplementary Figure S5. Pre-rRNA covers whole individual mitotic chromosomes. a**, Pre-rRNA covers individual chromosomes. The RNA signal was examined by RNA FISH using Cy5-labeled probe against 5'ETS region on mitotic spreads. The RNA FISH signal was analyzed by Local Thickness of Fiji to calculate the relative average extension distance of the 45S pre-rRNA. **b**, The mitotic spreads were treated with/without RNase A or RNase H. The fluorescence intensity of pre-rRNA and DAPI was measured at the indicated section with white lines, grey shadow shows the perichromosomal layer of pre-rRNAs (right panel). Scale bar: 5μm. **c**, The RNA FISH signal was analyzed by Local Thickness of Fiji, the dashed square shows the region of interest.

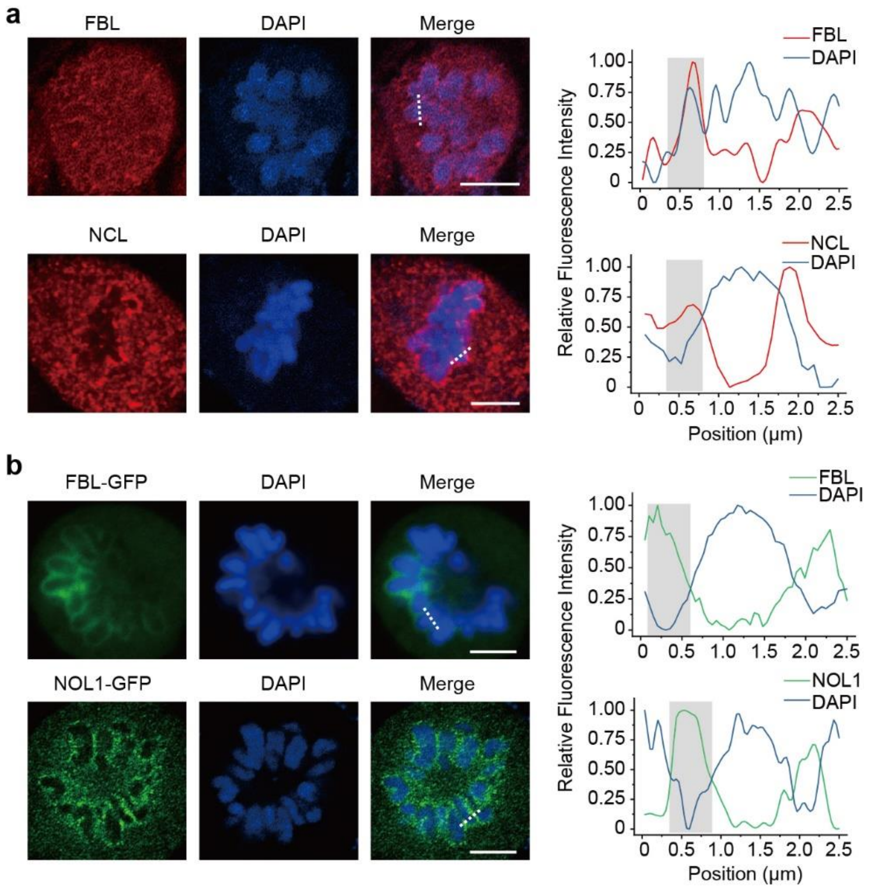


**Supplementary Figure S6. Nucleolar proteins localize at periphery of mitotic chromosomes. a**, The localization of FBL, NCL at the perichromosomal layer. FBL and NCL were immunostained with corresponding antibodies respectively. **b**, The localization of NOL1-GFP and FBL-GFP at the perichromosomal layer was detected. The fluorescence intensity of protein and DAPI was measured at the indicated section with white lines, grey shadow shows the perichromosomal layer of protein signal. Scale bar: 5μm.

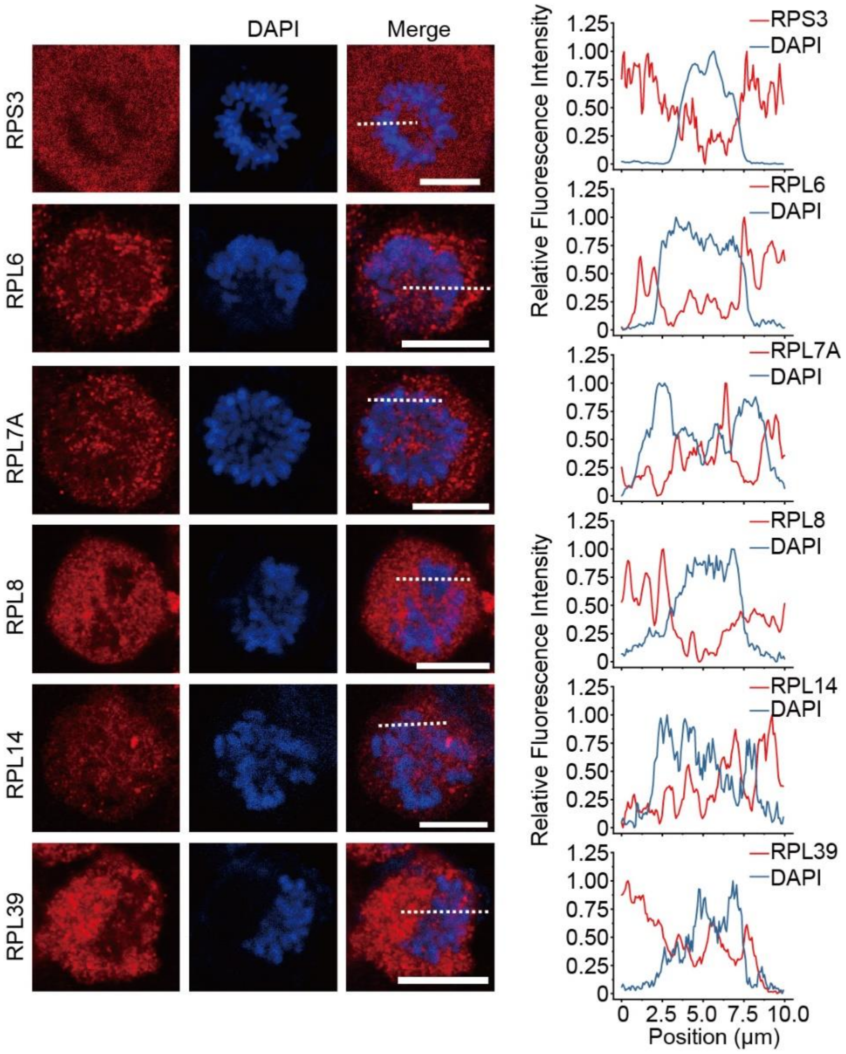


**Supplementary Figure S7. Ribosomal protein subunits do not localize at periphery of mitotic chromosomes.** The localization of RPS3, RPL6, RPL7A, RPL8, RPL14 and RPL39 were examined with corresponding antibodies. The fluorescence intensity of proteins and DAPI was measured at the indicated section with white lines. Scale bar: 10 μm.

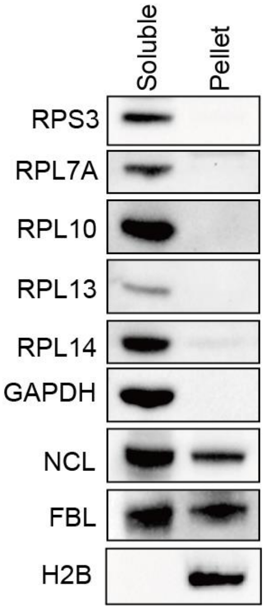


**Supplementary Figure S8. Ribosomal protein subunits do not exist in the pellets.**

The soluble and pellet fractions isolated from colcemid treated cells were examined by western blotting with indicated antibodies.

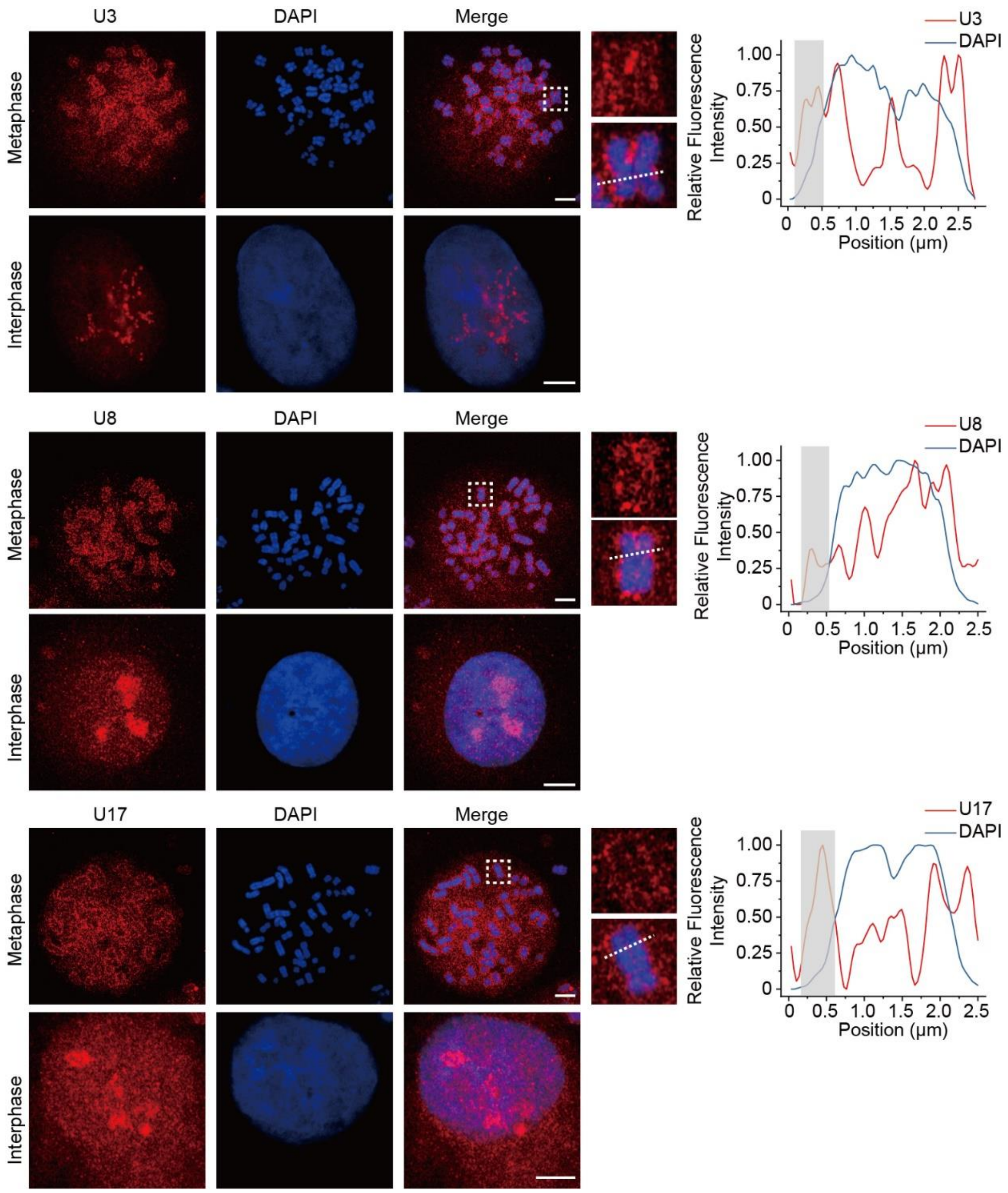


**Supplementary Figure S9. SnoRNAs localize at periphery of mitotic chromosomes.**

The localization of U3, U8 and U17 snoRNAs was detected by RNA FISH using Cy3-labeled probes against U3, U8 and U17 at the perichromosomal layer in mitotic spreads and nucleoli in interphase cells. The fluorescence intensity of RNA and DAPI was measured at the indicated section with white lines. Scale bar: 5μm.

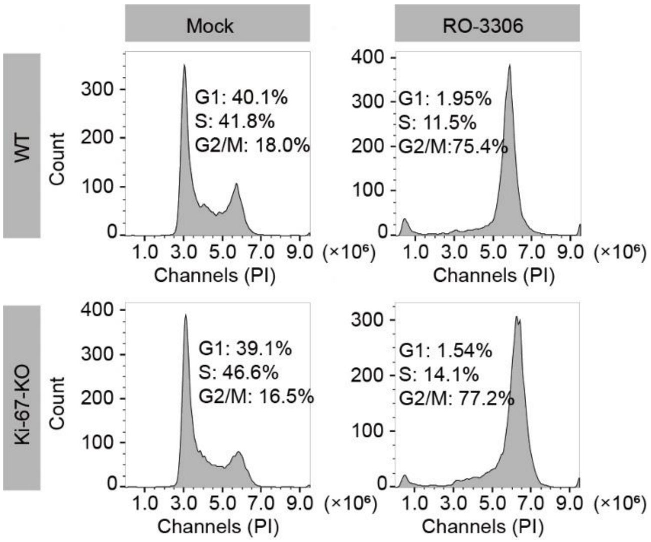


**Supplementary Figure S10. Cell cycle profiles following CDK1 inhibitor treatment.**

The cells were treated with the CDK1 inhibitor (RO-3306) for overnight.

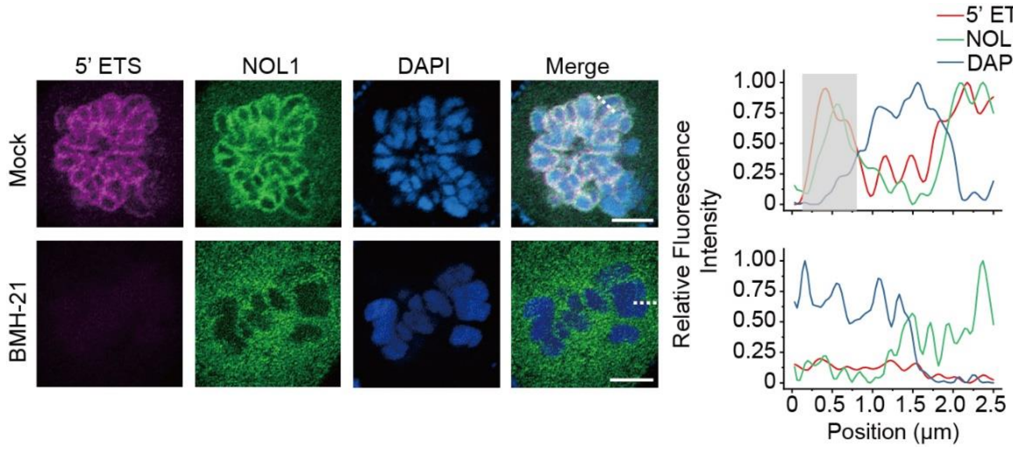


**Supplementary Figure S11. NOL1 localizes at periphery of mitotic chromosomes.** The localization of NOL1 was examined in the cells treated with or without BMH-21. The 45S pre-rRNA was examined by RNA FISH using Cy3-labeled probe against 5'ETS region of pre-rRNA, NOL1 was fused with an EGFP tag and detected. The fluorescence intensity of 45S pre-rRNA/NOL1 and DAPI was measured at the indicated section with white lines. Scale bar: 5μm.

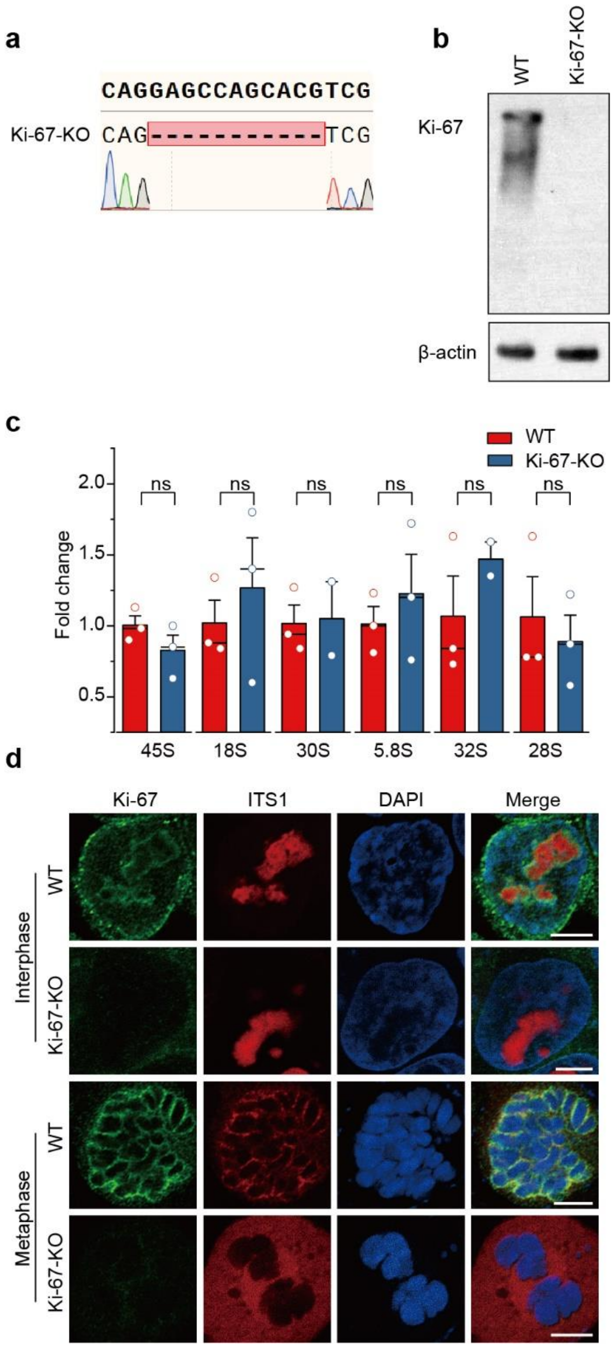


**Supplementary Figure S12. Loss of Ki-67 does not affect rRNA transcription.** Sanger sequencing analysis (**a**) and western blot (**b**) to validate the knockout of Ki-67 in 293T cells. **c**, qPCR analysis of rRNAs in parental 293T and Ki-67 knockout cells. ns: no significance. **d**, Loss of Ki-67 impairs the localization of pre-rRNA at the periphery of chromosomes. Ki-67 was immunostained with anti-Ki-67 antibody. Scale bar: 5μm.

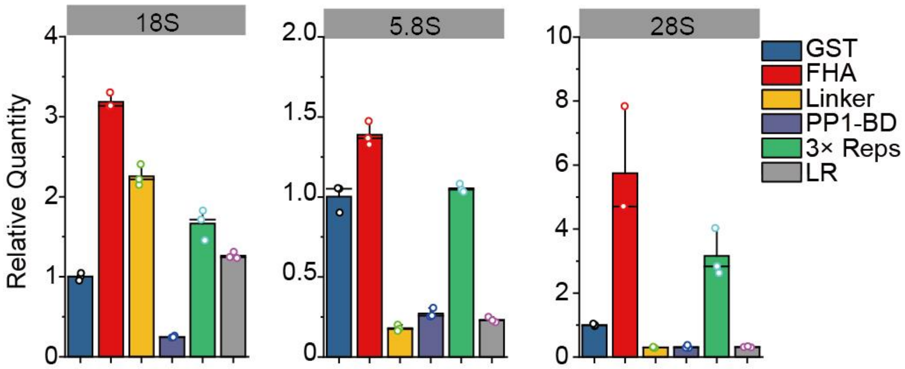


**Supplementary Figure S13. Mapping the domains of Ki-67 interacting with pre-rRNAs.** The analysis of qPCR with the primers for 18S, 5.8S and 28S rRNAs. The location of primers is shown in Supplementary Fig. S1e.

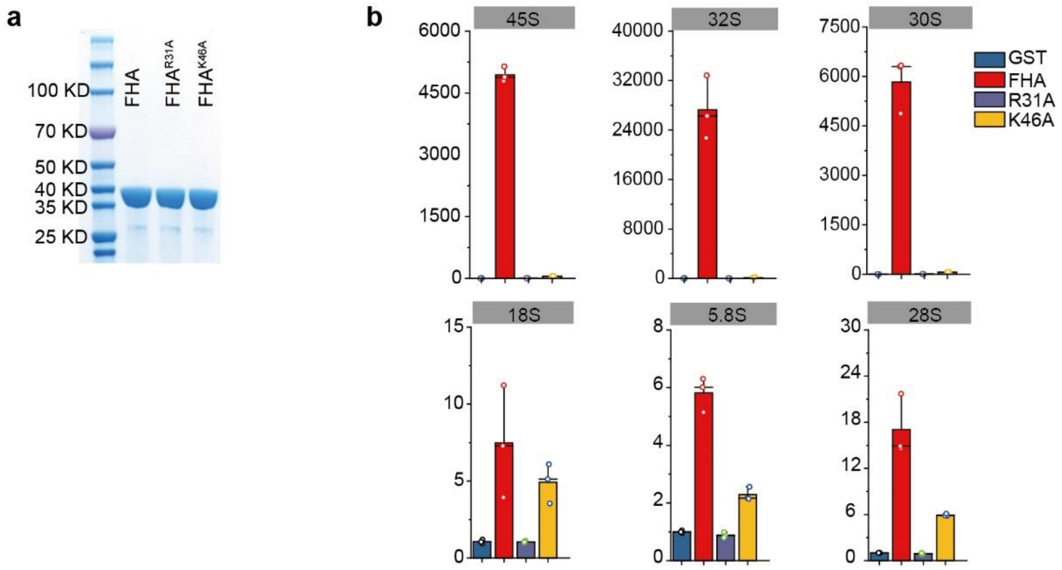


**Supplementary Figure S14. The FHA mutations of Ki-67 abolish the interaction with pre-rRNA. a**, Recombinant FHA, the R31A, K46A mutants were examined by Coomassie brilliant blue (CBB). **b**, Recombinant proteins were incubated with total RNA, then the RNAs pull-down by the proteins were extracted, followed by the analysis of RT-qPCR. The localization of primers is shown in Supplementary Fig. S1e.

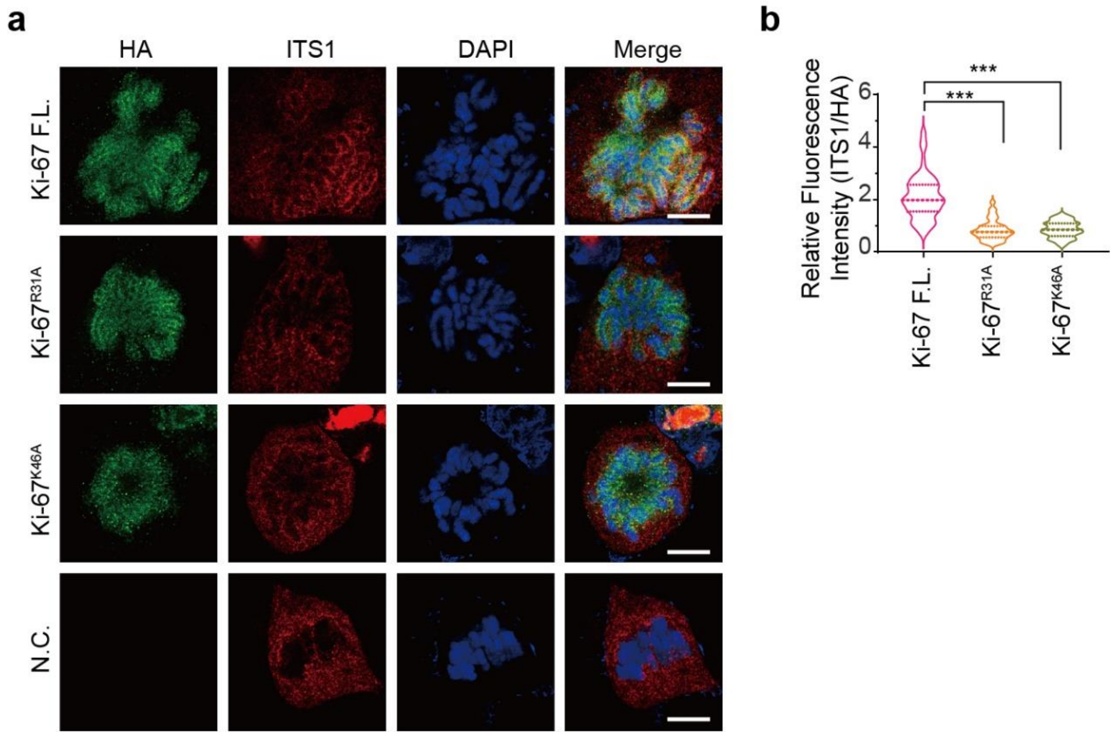


**Supplementary Figure S15. The FHA mutations of Ki-67 impairs loading of pre-rRNAs onto mitotic chromosomes. a**, The Ki-67-dificient cells were reconstituted with full-length or its mutants. RNA FISH was performed with Cy3-labeled probe against ITS1 region, HA tagged full-length and mutant Ki-67 was examined by anti-HA antibody. Scale bar: 5μm. **b**, Statistical analysis of the relative fluorescence intensity of ITS1 probe along the chromosomes is shown. The results were normalized by Ki-67 expression (the signal intensity of HA tag). ****P* < 0.001.

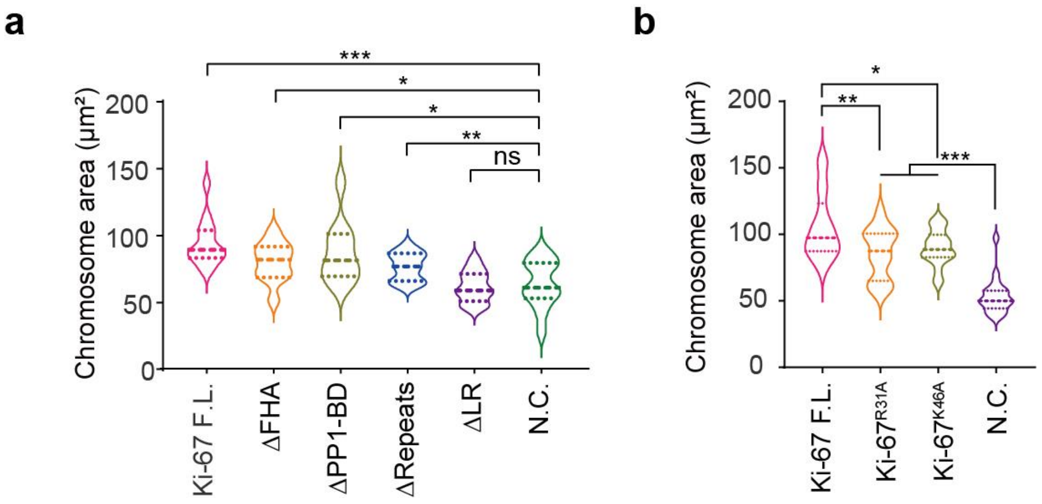


**Supplementary Figure S16. Pre-rRNA facilitates chromosome dispersion at prometaphase. a, b,** Ki-67 promotes chromosome dispersion. Chromosome areas at prometaphase were measured in the cells expressing full-length Ki-67 or truncation mutants (**a**) or point mutants (**b**). *$P < 0.05$; **$P < 0.01$; ***$P < 0.001$.

**Supplementary Table 2. Sequences of oligonucleotides used as primers in RT-qPCR and probes in northern blot assays**

| Oligo probe name | RT-qPCR primers |
|---|---|
| 45S-qPCR-F | CCCACCCTCGGTGAGAAAAG |
| 45S-qPCR-R | GGAAGCGGAGGAGGGTCCTC |
| 18S-qPCR-F | GGCCCGAAGCGTTTACTTTG |
| 18S-qPCR-R | GCGGCGCAATACGAATGCC |
| ITS1-qPCR-F | CGAGAGCCGGAGAACTCGG |
| ITS1-qPCR-R | GCCGACACCCACGTCGTC |
| ITS2-qPCR-F | CGGGCCCTGCGTGGTCAC |
| ITS2-qPCR-R | GGAGGAACCCGGACCGCAG |
| 28S-qPCR-F | CACGAGACCGATAGTCAACAAG |
| 28S-qPCR-R | AACGGGGGGCGGGAAAGATC |
| 5.8S-qPCR-F | GACTCTTAGCGGTGGATCAC |
| 5.8S-qPCR-R | AAGCGACGCTCAGACAGGC |

| Oligo probe name | Northern blot probes |
|---|---|
| 28S-Biotin | AACGATCAGAGTAGTGGTATTTCACC |
| 18S-Biotin | ACGGCGACTACCATCGAAAG |
| 5.8S-Biotin | GTTCTTCATCGACGCACGAG |
| ITS1-Biotin | CCTCGCCCTCCGGGCTCCGTTAATGATC |
| ITS2-Biotin | CTGCGAGGGAACCCCCAGCCGCGCA |

**Supplementary Table 3. Sequences of oligonucleotides used as probes in RNA FISH assays**

| Custom Stellaris RNA FISH probes (FAM linked) | |
|---|---|
| **No.** | **ITS1 probe (human)** |
| 1 | TCGCCCTCCGGGCTCCGT |
| 2 | TGGGTGTGCGGAGGGAAG |
| 3 | CGAACGAACGGCACGCG |
| 4 | GGGCGGCGAACGAACGAG |
| 5 | CGAGTTCTCCGGCTCTCG |
| 6 | TCTCTCTCTCTCTCTCTC |
| 7 | GACACGCCCTTCTTTCTC |
| 8 | ACGACACGCGCACACCAA |
| 9 | GGAGGAGGGCACCGAGAC |
| 10 | GCGGAGGCGACGGGAATC |
| 11 | AGACGCCCTAGCGGGAAG |
| 12 | GACGAGGAGGCGGGGGAG |
| 13 | GAACGCGCTAGGTACCTG |
| 14 | CCCCAAGGGGTCTTTAAA |
| 15 | TTTCACACCACGGGGAGG |
| 16 | CCGGAGAGGGGTCGGAAG |
| 17 | GAGACAGCGAACGGGACC |
| 18 | CCAAGAGGAGAGGGGGTT |
| 19 | ACGACGCACCGGGAGGAG |
| 20 | GAGGTCGATTTGGCGAGG |
| 21 | ACAGGCGCCCGGGGGTTC |
| 22 | CACGCGCCGCGTCGCGGT |
| 23 | GCCGACACCCACGTCGTC |
| 24 | CGCGACGCCGCCGAGAAC |
| 25 | GAAGACGGGGAGCCGGCG |
| 26 | AGAGCGAGCGGGGCCGTG |
| 27 | CCCGACCCACGGGCGGAC |
| 28 | CCCTCCCGACGGGACTCC |
| 29 | AGTCCGCGGTGGAGGCGC |
| 30 | GGGTAAAGCCCCCACCCGAC |
| 31 | CACGCCACACGCGCGGCA |
| 32 | GGGGGCGAGCGCGGACAC |
| 33 | CTTCCTGGCGCGGCACGT |

| Other RNA FISH probes | |
|---|---|
| **Name** | **RNA FISH probes (human)** |
| 45S-Cy5 | ACGACGTCACCACATCGATCACGAAGAG |
| ITS1-Cy3 | CCTCGCCCTCCGGGCTCCGTTAATGATC |
| ITS2-Cy3 | CTGCGAGGGAACCCCCAGCCGCGCA |
| 28S-Cy3 | CCTTGTGTCGAGGGCTGACTTTCAATAG |
| 18S-Cy3 | TTTACTTCCTCTAGATAGTCAAGTTCGACC |
| U3-Cy3 | GCTTCACGCTCAGGAGAAAACGCTACCTC |
| U17-Cy3 | GGCTGTTTCCTGCATGGTTTGTCTCCCCAG |
| U85-Cy3 | CTTAGCCAAACCAACTGAATCACAACAGCCTTGATATCATCATGTG |
| NEAT1-Cy3 | CTGCTGGCATGGACAAGTTGAAGATTAG |
| ITS1-sense-Cy3 | GATCATTAACGGAGCCCGGAGGGCGAGG |
| ITS2-sense-Cy3 | TGCGCGGCTGGGGGTTCCCTCGCAG |
| **Name** | **RNA FISH probes (mouse)** |
| 18S-Cy3 | GAGGTTTCCCGTGTTGAGTCAAATTAAGCCGCA |
| 28S-Cy3 | ACGGGCTGGGCCTCGATCAGAAGGACTTGG |
| 5.8S-Cy3 | CATCGACGCACGAGCCGAGTGATCCAC |
| ITS1-Cy3 | ACGGGTCAAAAACCCGTAACGACGTA |
| ITS2-Cy3 | ACCGCCCACACGTCTGAACTTCGGGAGAC |

GAPDH **SMF-2026-1** Stellaris® FISH Probes, Human GAPDH with Quasar® 570 Dye
